# Aging science comes of age

**DOI:** 10.1038/npjamd.2015.7

**Published:** 2015-09-28

**Authors:** Kazuo Tsubota

**Affiliations:** 1 Editor-in-Chief, President, Japanese Society of Anti-Aging Medicine, Departments of Ophthalmology and Health Science Laboratory, Keio University School of Medicine, Shinjuku-ku, Tokyo, Japan

Aging is at the center of many of the world’s most prevalent and deadly diseases, including cancer and heart disease. Over the last 20 years we have seen amazing advances in our understating of the mechanisms behind aging-related processes at the level of genes, cells and whole organisms. Many countries are now facing a growing aging society, with a high prevalence of fatal age-related diseases, such as cancer, cardiovascular, and pulmonary disease.

Exciting new lines of research have shown that aging involves a complex interplay between the genome, epigenome, microbiome, and the environment. Recent work on epigenetics modifications, the chemical signatures branded on our genome that affect gene expression, has revealed surprising links to the aging process and established a connection with environmental factors.^1^ Important epigenetic marks such as the methylation of regulatory DNA sequences, covalent modifications of histone proteins, and the expression of regulatory non-coding RNAs are affected during aging.

The effect of the environment on this epigenetic landscape is clearly shown by studies using identical twins. As they age, these twins are no longer identical in their epigenome, showing differences in gene expression and ultimately, lifespan.^2^ Changes to the epigenetic landscape may affect gene expression and ultimately the aging process, especially through the modification of metabolism.

Food scarcity is one of the most important environmental factors affecting epigenetic modification; it is therefore not surprising that many molecules and signaling pathways that have been implicated in aging, such as mammalian target of rapamycin, sirtuins, and insulin-like growth factor/insulin signaling pathways, are related to metabolism. Thus, how our genes modify our metabolic status, depending on the food intake and consumption, is a central issue of aging science.

Another new and exciting area in aging research involves the tiny microbes living in our bodies. Our gut, for example, hosts some 1×10^13^ to 1×10^14^ microorganisms that amount for 10 times as many cells than found in our body and 150 times as many genes as there are in our genome. Recent work has revealed the influence of these microorganisms that have co-evolved with us for more than 500 million years in multiple physiological processes, from the development and function of immune responses to the regulation of nutrient absorption, fat distribution, to neurological processes and more recently to the process of aging.^3^

Yet another nascent and exciting area in aging research involves the role that light plays in our circadian rhythms. The circadian clock has been linked to various physiological and behavioural processes, including energy metabolism, gastrointestinal tract motility, sleep-wake cycles, cardiovascular activity, endocrine secretion, body temperature, renal activity, and locomotor activity.^4^ Disrupting this clock has profound effects on our sleep patterns, and can lead to hypertensive crises, myocardial infarction, asthma and allergy attacks, and can even affect our mood and learning ability. Recent research has revealed interplay between excessive light and circadian rhythms and the activity of fat burning tissue. In a recent report, mice exposed to light beyond their usual light cycle ended up gaining more fat than mice exposed to normal light/dark cycles.^5^ Age-related deregulation of the circadian clock affects the function of specific genes that in turn may lead to age-related diseases. For instance, mice in which genes linked to the circadian clock are knocked out suffer multiple life-shortening conditions, such as cardiac hypertrophy and epilepsy.^6^ The findings are a reminder of the importance of maintaining healthy circadian rhythms, and future research in this area warrants exciting findings with important clinical applications.

Sensory input and our mental condition are also considered to play a role in healthy longevity. Cross sectional and longitudinal epidemiological studies have found support for the commonly held belief that ‘happy people live longer!’.^7,8^ Recent intervention studies as well as animal studies, such as environmental enrichment, provide further support for this concept.^9^

Understanding the fundamental mechanisms of aging may lead to the development of new treatments that could be applicable to a wide variety of age-related diseases. *npj Aging and Mechanisms of Disease*—our new open access journal—provides a forum for the world’s leading researchers in the field of aging and age-related diseases. The journal will bring together research aimed at elucidating the mechanisms behind aging and age-related disorders, including research in mitochondrial metabolism, DNA damage response, oxidative stress, circadian rhythm, epigenetics, autophagy, and the microbiome.

We invite submissions on new approaches to treatment of age-related diseases, such as caloric restriction mimetics, practical exercise approach lifestyle modification for circadian disruption and on cancer prevention. *npj Aging and Mechanisms of Disease* seeks to attract articles from basic researchers, as well as clinicians who wish to apply aging-related findings to their practice. We look forward to your contributions.
